# Adjoint method-based Fourier neural operator surrogate solver for wavefront shaping in tunable metasurfaces

**DOI:** 10.1016/j.isci.2024.111545

**Published:** 2024-12-06

**Authors:** Chanik Kang, Joonhyuk Seo, Ikbeom Jang, Haejun Chung

**Affiliations:** 1 Department of Artificial Intelligence, Hanyang University, Seoul 04763, South Korea; 2 Department of Electronic Engineering, Hanyang University, Seoul 04763, South Korea; 3 Department of Computer Engineering, Hankuk University of Foreign Studies, Yongin 17035, South Korea

**Keywords:** Physics, Optics, Computer science

## Abstract

We present a Fourier neural operator (FNO)-based surrogate solver for the efficient optimization of wavefronts in tunable metasurface controls. Existing methods, including the Gerchberg-Saxton algorithm and the adjoint optimization, are often computationally demanding due to their iterative processes, which require numerical simulations at each step. Our surrogate solver overcomes this limitation by providing highly accurate gradient estimations with respect to changes in tunable meta-atoms without the need for direct simulations. This approach substantially reduces both computational time and cost in wavefront shaping applications. The proposed solver demonstrates a residual of 0.02 when compared to the normalized figure of merit achieved by the optimized structure obtained through the adjoint method, and its inference time is 887.5 times faster than conventional simulation-based methods. This advancement enables ultra-fast wavefront shaping across a range of applications, including optical wavefront shaping, reconfigurable intelligent metasurfaces, and biomedical imaging.

## Highlights

• Surrogate solver predicts adjoint gradients without full-wave simulations
• FNO-based solver replaces iterative adjoint method for fast wavefront shaping
• Our method accelerates metasurface optimization by 887.5 times with high accuracy
• Enables rapid, simulation-free wavefront control in tunable metasurfaces

## Graphical abstract

**Figure:**
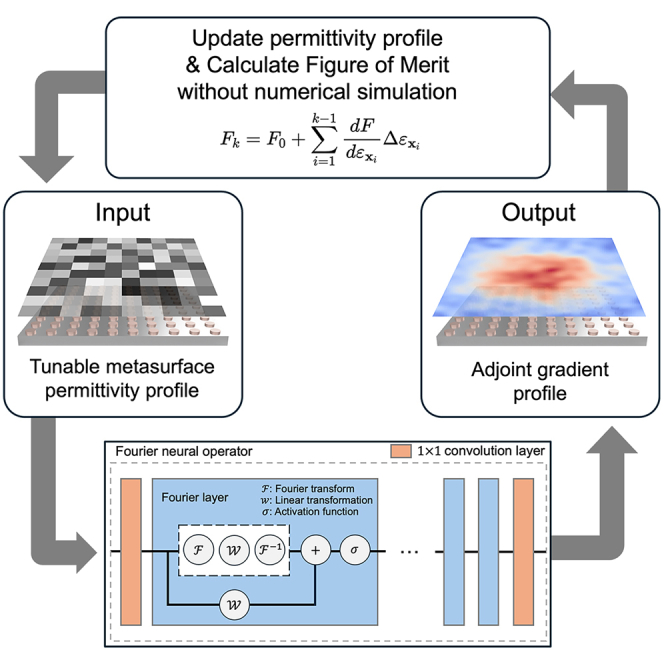

## Introduction

Metasurfaces have garnered significant attention for their ability to achieve complex functionalities beyond those of traditional optical components.^1^^,^^2^ Notably, they have enabled the development of ultra-thin lenses with high numerical apertures.^3^^,^^4^ Recent advancements in simulation and machine learning techniques have also facilitated the efficient design of metastructures,^5^^,^^6^^,^^7^ further enabling the creation of highly efficient holographic devices with precise image reconstruction and minimal loss.^8^ Recent studies have also demonstrated that metasurfaces can achieve near-perfect efficiency in beam steering applications.^9^^,^^10^^,^^11^ Unlike passive metasurfaces,^12^ tunable metasurfaces^10^^,^^13^ can dynamically adjust their optical properties in response to external stimuli such as electrical signals,^14^^,^^15^^,^^16^ mechanical stress,^17^ or temperature variations.^18^ Wavefront shaping with tunable metasurfaces^19^^,^^20^ have contributed to diverse applications such as holography,^21^^,^^22^ biomedical imaging,^23^ beam steering,^24^ and beam focusing.^25^ Achieving the desired wavefront requires precise optimization of the metasurface structure or the effective permittivity profile in the case of tunable metasurfaces. One widely adopted method for wavefront shaping is the Gerchberg-Saxton (GS) algorithm,^26^^,^^27^^,^^28^ which iteratively minimizes the error between the target and current wavefronts by employing Fourier transform (FT) and inverse FT (IFT) processes. Another approach involves the feedback loop method, which optimizes design parameters through a real-time feedback system.^29^^,^^30^

Conventional optimization methods for wavefront shaping are inherently iterative, requiring numerical simulations at each step. As the design space expands, the computational resources and simulation timescale accordingly. While these conventional methods remain viable for many metasurface studies, transitioning to large-scale wavefront shaping requires approaches capable of achieving comparable results within a given computational capacity. One way of sidestepping this issue is the adjoint method,^31^ a gradient-based inverse design technique that efficiently calculates the gradient of an objective function concerning the design parameters. This method requires only two full-wave simulations. One is for the forward simulation, and one is for the adjoint (backward) simulation per iteration, significantly reducing the computational cost compared to traditional optimization methods that require multiple forward simulations for gradient calculation. The adjoint method calculates the gradient of a vector objective function *F* with respect to the permittivity distribution ϵ(x) using only two simulations per iteration, utilizing Born approximation^32^ and Lorentz reciprocity.^33^ This is achieved through the formula^31^:

$$\frac{\partial F}{\partial \epsilon \left(x\right)}=\text{Re}\left[E_{\text{dir}\;}\left(x\right)\cdot E_{\text{adj}\;}\left(x\right)\right]$$

**Figure 1:**
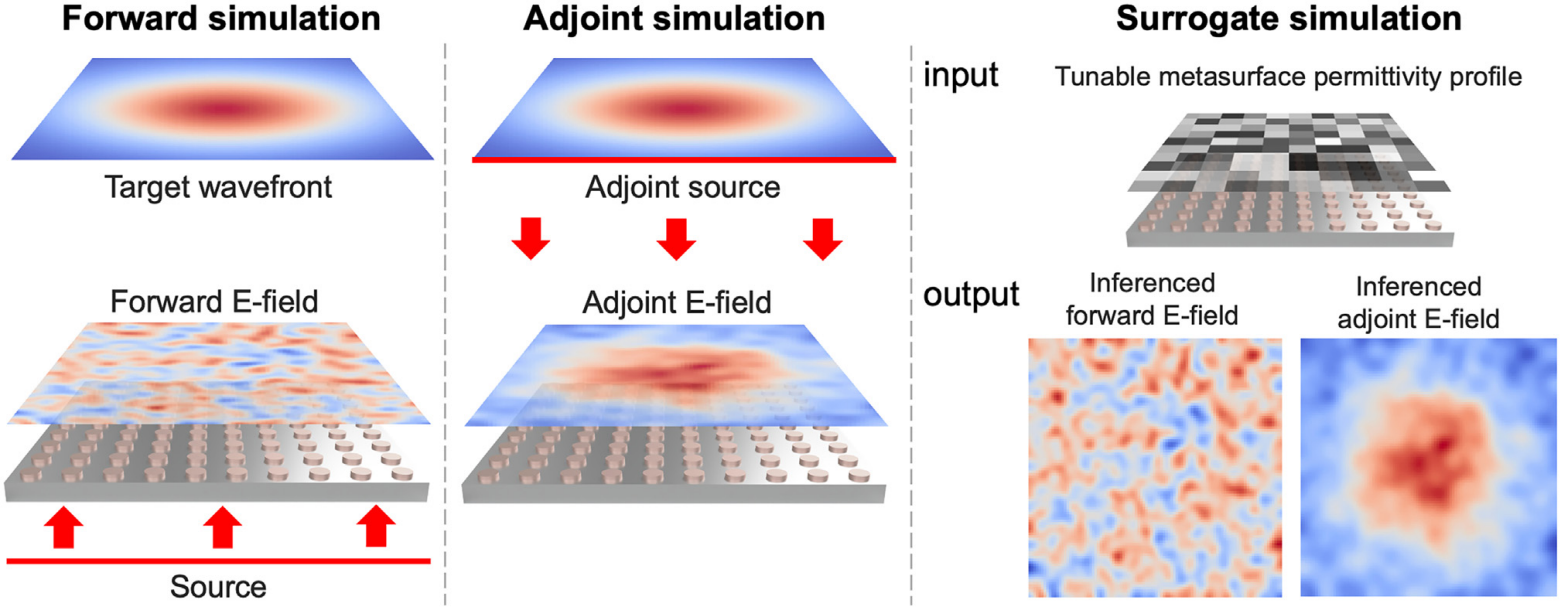
Overview of forward simulation, adjoint simulation, and surrogate simulation In the forward simulation, the source wave propagates through the tunable metasurface, where each liquid crystal (LC)-based meta-atom can be adjusted by altering the LC director, thereby modifying the permittivity tensor. The resulting forward electric field is recorded for subsequent gradient calculations. At the target plane, the figure of merit is determined by comparing the simulated wavefront to the desired wavefront. This comparison leads to the derivation of the adjoint source ($J_{\text{adj}}=-i\omega P_{\text{adj}}=-i\omega \partial F/\partial E$), where P is a source dipole density.^31^ The adjoint source backpropagates toward the meta-atoms, and the adjoint gradient is calculated by taking the dot product of the electric fields from both the forward and adjoint simulations. In contrast, the surrogate solver approach allows the adjoint gradients to be predicted by a trained neural network, significantly speeding up the optimization process.

$E_{\text{dir}\;}\left(x\right)$ represents the electric field obtained from the forward simulation. In contrast, $E_{\text{adj}\;}\left(x\right)$ corresponds to the electric field from the adjoint (backward) simulation.^31^ The dot product of these fields, followed by taking the real part, yields the adjoint gradient, which indicates how changes in the permittivity ϵ(x) at a given position x will affect the objective function. As illustrated in Figure 1, adjoint optimization requires both a forward and an adjoint simulation to compute the adjoint gradient. These two simulations enable the acquisition of the electric fields within the design region, which are used to calculate the adjoint gradient for the optimization. However, despite the computational efficiency of the method, it still requires two full-wave simulations per iteration, posing significant challenges in large-scale problems due to the associated computational demands.

To address this issue, we propose a new approach that maintains the core optimization principles of the adjoint method while inferring gradients without the need for extensive numerical simulations. Specifically, we introduce an adjoint method-based Fourier neural operator (FNO) surrogate solver. The FNO-based surrogate solver has been widely used in fields such as computational fluid dynamics^34^^,^^35^ and computer-aided engineering-based studies.^36^^,^^37^ The approach is appealing due to the physically reasonable property of discretization invariance,^38^ which potentially offers mesh-independent inference of the partial differential equations (PDEs) solutions through learning mappings between function spaces.^39^ In other words, learning mappings between function spaces implies that the neural operator is designed to approximate operators that map input functions to output functions rather than just mapping finite-dimensional vectors as in traditional neural networks. This functional perspective allows the neural operator to generalize across different discretizations and mesh resolutions, enabling mesh-independent predictions. Neural operators, such as the FNO, can learn PDEs by approximating the solution operator of PDEs, effectively capturing the underlying functional relationship between input conditions and the PDEs solutions. By leveraging global kernels and spectral methods like the FT, the FNO efficiently models complex dependencies inherent in PDEs, facilitating accurate and efficient inference without the need for extensive numerical simulations.^40^^,^^41^

For surrogate solvers to be widely adopted in optics and photonics, we propose a solver that can predict adjoint gradients without numerical simulations, which may significantly reduce the computational cost of the large-scale inverse design. The main contributions of this study include the development of a surrogate solver aimed at significantly enhancing the speed of adjoint optimization in photonics, specifically for optimizing the permittivity profile of a tunable metasurface to generate a desired wavefront. The proposed solver accurately predicts adjoint gradients with a mean absolute error (MAE) of less than 0.02 compared to numerically calculated adjoint gradients. Additionally, it achieves an inference speed of 887.5 times faster than the full-wave adjoint method, enabling ultra-fast wavefront shaping. The performance of this surrogate solver surpasses that of conventional techniques such as the GS algorithm, feedback loops, and the adjoint method itself.

## Results and discussion

### Adjoint-based Fourier neural operator

**Figure 2:**
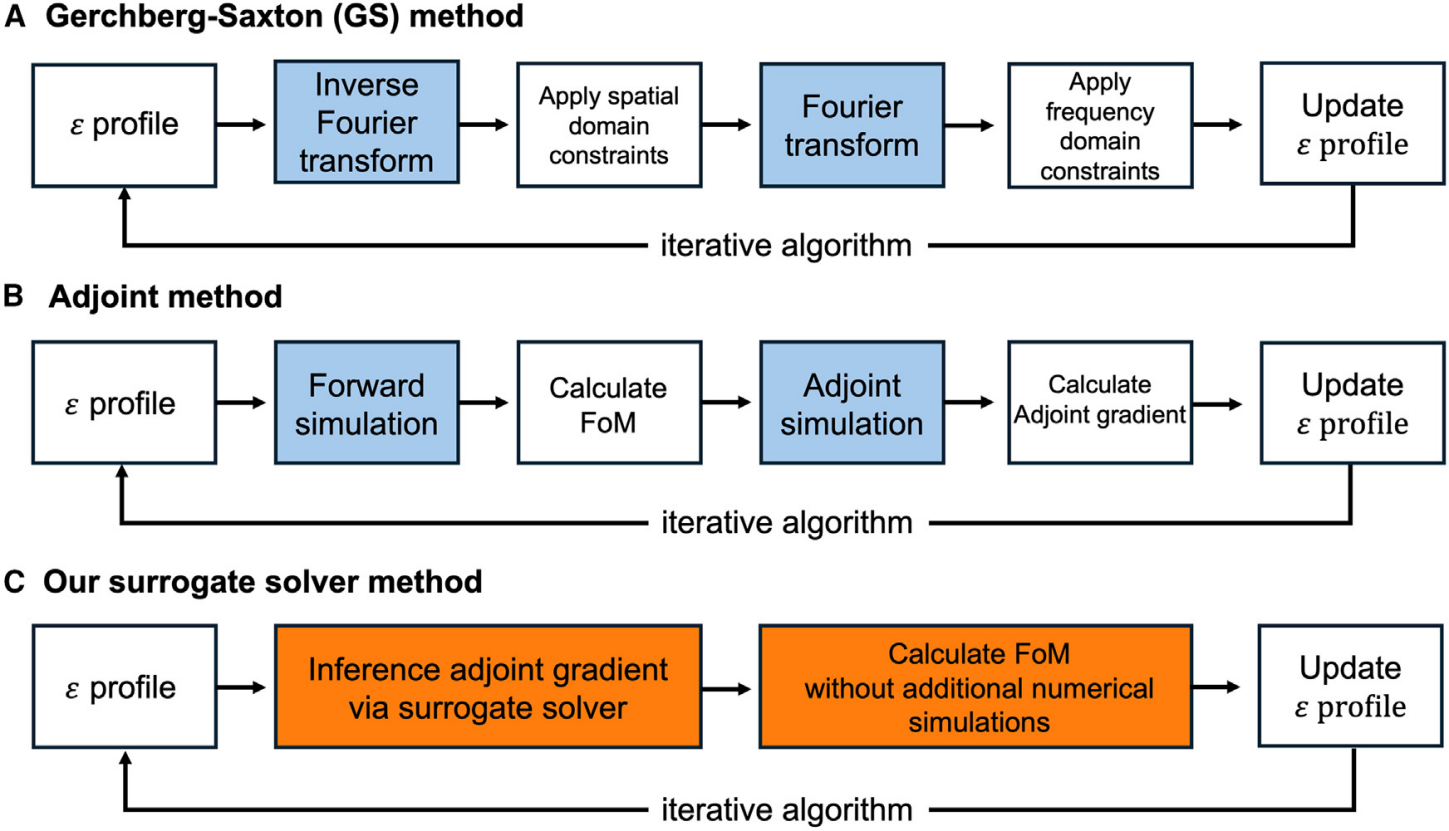
Wavefront shaping methodologies (A) The Gerchberg-Saxton method involves one inverse Fourier transform (IFT) and one Fourier transform (FT) in each iteration. (B) In comparison, the adjoint method requires one forward and one adjoint simulation per iteration. (C) Alternatively, the surrogate solver quickly infers the adjoint gradient without performing numerical simulations and can predict changes in the figure of merit (FoM) with respect to the changes in the design parameters (e.g., permittivity tensor), eliminating the need for additional simulations.

The problem we address is the optimization of the effective permittivity profile of meta-atoms in a tunable metasurface to achieve a desired wavefront. Specifically, we focus on a 2D wavefront shaping problem in the transverse electric (TE) mode, where the liquid crystal (LC) permittivity tensor is simplified to $\varepsilon_{zz}$.^13^ Furthermore, we assume that each LC-based meta-atom has a uniform permittivity within the unit cell, neglecting coupling effects with neighboring meta-atoms and disregarding any misalignment of the LC director. One approach to solving this problem is the GS method, illustrated in Figure 2A. While this method is relatively straightforward and effective for phase retrieval, it suffers from slow convergence and is highly sensitive to initial conditions, rendering it less suitable for large-scale optimization problems.^42^ One alternative is the adjoint method. As demonstrated in Figure 2B, the adjoint method can effectively reduce the number of simulations required for optimization. However, its computational cost scales with the size of the optimization problem.^43^

To reduce computational burden and time demands, we propose a surrogate solver based on FNO, which replaces the numerical simulations required in the adjoint method while ensuring physically plausible outcomes. Once trained, the FNO-based surrogate solver, shown in Figure 2C, demonstrates ultra-fast inference times and delivers highly accurate results, regardless of the problem’s size.

Similar to the adjoint method, our approach calculates the adjoint gradient for the design region by taking the dot product of the electric fields (E-fields) obtained from both forward and adjoint (inverse) simulations. While both methods yield the same adjoint gradient, their computational processes differ. As illustrated in Figure 2B, the adjoint method requires two full-wave simulations—one for the forward and another for the adjoint simulation—to compute the necessary E-fields. In contrast, our surrogate solver, powered by the FNO, computes the adjoint gradient with minimal error relative to the adjoint method. As depicted in Figure 2C, the surrogate solver predicts performance variations based on the gradient and permittivity changes, eliminating the need for additional numerical simulations. It accurately computes the gradients needed for optimization by reliably predicting both the forward and adjoint fields.

The method for predicting the figure of merit (FoM) of an updated structure without additional numerical simulations is described in the “performance metric calculation without numerical simulations” section.

### Experiments results

**Figure 3:**
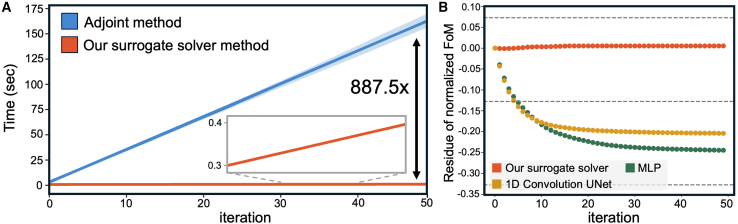
Comparison of computation speed and inference accuracy between the adjoint method and our surrogate solver (A) Iteration count versus simulation time for finite-difference time-domain (FDTD)-based adjoint optimization compared to our proposed surrogate method. (B) Inference accuracy of our surrogate solver compared to an MLP and a 1D convolutional UNet. The y axis shows the residue, which is the difference between the normalized figure of merit (FoM) from surrogate methods and the FoM from FDTD-based adjoint optimization. A residue near zero indicates that the surrogate-optimized structure closely matches the FDTD-optimized structure. Our method achieves a residue close to zero, showing comparable performance to FDTD-based optimization.

To validate our surrogate solver as a viable alternative to traditional methods, we evaluate three key aspects: speed, accuracy, and optimization performance. As shown in Figure 3A, the adjoint method is significantly accelerated by our trained surrogate solver, which infers gradients almost instantaneously with minimal computational time, demonstrating a substantial improvement in speed.

As the number of iterations increases, the difference in computation time becomes increasingly pronounced. In our experimental setup, after 50 iterations, the computation time is reduced by a factor of approximately 887.5.

Second, in terms of accuracy, our surrogate solver infers adjoint gradients that are nearly identical to those obtained using the traditional adjoint method. Figure 3B illustrates a comparison of the residue between the simulated (normalized) FoM and the FoM predicted by various models. The normalized FoM refers to the FoM value scaled to [0,1] based on simulated FoM values, as follows:

$$\text{Normalized}\;\text{FoM}\left(x\right)={\text{FoM}}_{\text{pred}}\left(x\right)/{\text{FoM}}_{\text{sim}}\left(x_{\text{final}}\right)$$

where *x* denotes the permittivity profile of the meta-atoms. $x_{\text{final}}$ refers to the FoM of the structure at the final iteration when optimized using the adjoint method. The adjoint gradient values predicted by our surrogate solver exhibit a MAE close to zero when compared to the ground truth gradient profiles obtained through the adjoint method. This result indicates that the surrogate solver can significantly accelerate the adjoint optimization process. We also compare our method with multi-layer perceptron (MLP) and 1D convolutional UNet models, as shown in Figure 3B. Both the MLP and UNet models fail to predict adjoint gradients over multiple iterations accurately. This suggests that neural operators-based models may effectively learn function spaces while other models, such as discriminative or generative models, struggle to capture the adjoint gradient derived from the full-wave solutions of Maxwell’s equations.

**Figure 4:**
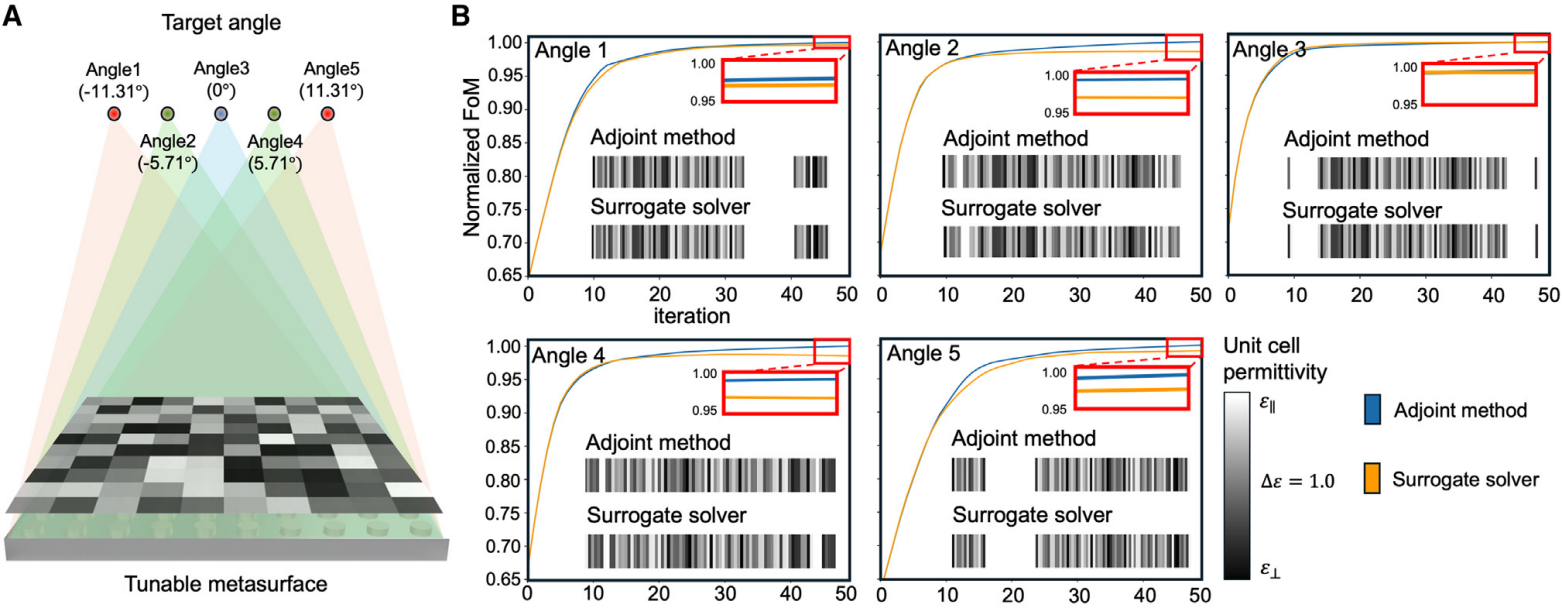
Comparison of optimization results based on beam-focusing target point angles (A) Schematic illustration of the LC-based tunable metasurface and the desired wavefront for beam focusing. The permittivity tensor of the meta-atom is assumed to vary with the application of external voltage to the LC. (B) Wavefront shaping of the tunable metasurfaces utilizing adjoint gradients. While the adjoint method efficiently discovers optimal metasurface configurations, it requires full-wave simulations of the entire region. In contrast, the surrogate solver approach achieves near-optimal metasurface configurations 887.5 times faster than the adjoint method. The inset shows the 2D permittivity profile of the meta-atoms.

Third, to evaluate the optimization accuracy, we conducted experiments to assess how effectively the tunable metasurface focuses the beam at the desired angles, as illustrated in Figure 4A. Figure 4B presents the optimization results for the five angles we set. The MAE of the optimization results from our method consistently remained below 0.02 when compared to the adjoint method. Given that our model predicts adjoint gradients nearly identical to those of the adjoint method, as shown in the second evaluation criterion, we verified whether the optimized structure at the final iteration, where the FoM converges, was the same for both methods. As shown in Figure 4C, the results produced by our surrogate solver are nearly identical to those obtained using the adjoint method, confirming the effectiveness of our approach.

**Figure 5:**
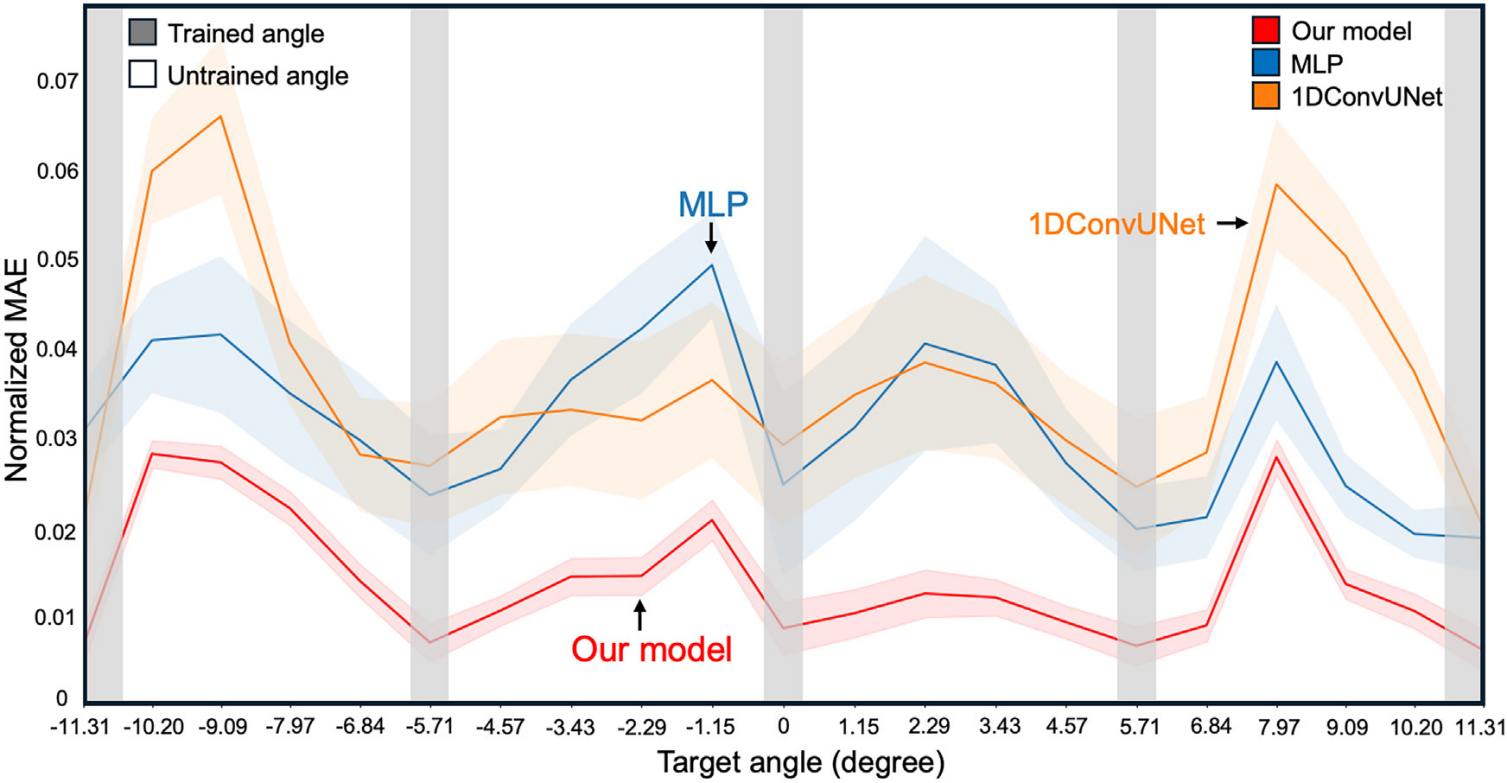
Adjoint gradient inferred by the surrogate solver for untrained conditions Experiments were conducted to evaluate the accuracy of the surrogate solver in predicting adjoint gradients for angles not included in the training dataset (0°, ±5.73°, and ±11.31°). The surrogate solver achieved the lowest normalized mean absolute error (MAE) across all intermediate angles, demonstrating its robustness in untrained conditions.

Additionally, we conducted experiments to evaluate whether our model can accurately predict adjoint gradients for intermediate (untrained) angles beyond those used in the training for beam focusing. If the model can only predict adjoint gradients for the specific angles it was trained on, and fails to generalize to untrained angles, this would necessitate generating and training on data for every angle, thereby limiting its versatility. As illustrated in Figure 5, we trained the model on five specific angles and tested its ability to predict adjoint gradient values for 16 interpolated angles between these trained points. We also compared our method to the MLP and 1D convolutional UNet models used in the accuracy comparison in Figure 3B. The results demonstrate that our surrogate solver achieved the lowest normalized MAE across all angles, highlighting its ability to infer adjoint gradients even for untrained angles.

In summary, our surrogate solver accurately predicts adjoint gradients while significantly reducing computational time. Moreover, it demonstrates the capability to infer adjoint gradients for untrained angles, making it a generalized solution for optimizing tunable metasurfaces. This capability is validated through the experiments presented in Figures 3, 4, and 5.

### Model establishment

**Figure 6:**
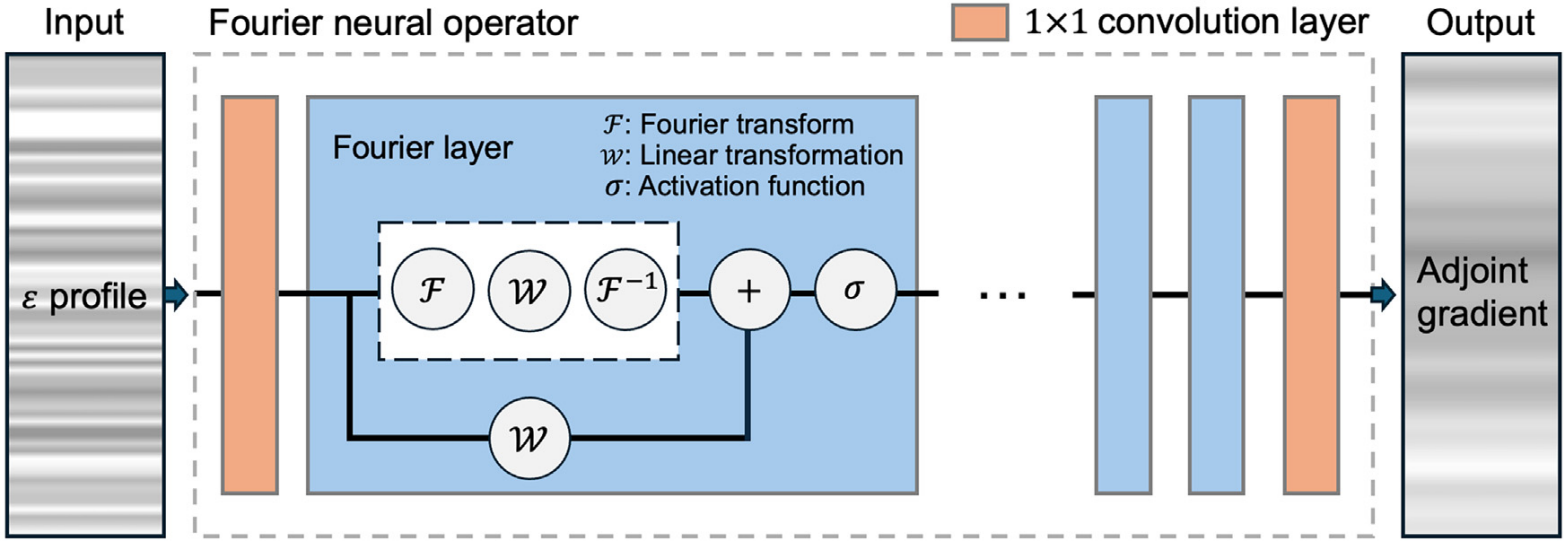
Schematic of our surrogate solver model The model accepts the permittivity profile of the tunable metasurface as input and outputs the corresponding adjoint gradient. For inference, we utilize the Fourier neural operator, leveraging multiple Fourier layers to effectively learn the underlying function space.

The schematic of our surrogate solver model is described in Figure 6. We define Ω∈R as the range of light propagation angles, bounded by [−ω,ω]. From this, $\tilde{\Omega }\subset \Omega$ represents a discrete subset of angles, sampled evenly at specific intervals.

Our goal here is to model an ideal adjoint optimizer $\Phi^{*}$ for continuous angles in Ω using the proposed model $\Phi_{\theta }$. This model is trained on a finite set of pairs, ${\left\{{\tilde{\omega }}_{i},a_{i}\left(x\right),u_{i}\left(x\right)\right\}}_{i=1}^{N}$, where $\tilde{\omega }\in \tilde{\Omega },a\left(x\right)\in R^{d_{\text{in}}}$ denote input permittivity profiles in the metasurface, and $u\left(x\right)\in R^{d_{\text{out}}}$ denote the corresponding adjoint gradients. The simulation data from PDE solver depends on the determined mesh information, therefore, we use *x* as a location on the simulation mesh. *x* referes to spatial coordinates where permittivity and gradients are computed. We employ the FNO^40^ to learn the mapping between function spaces. FNO operates iteratively, enabling the mapping of infinite-dimensional function spaces as follows:

$$u=\Phi_{\theta }\left(a\right)=\left(Q\circ K^{\left(L\right)}\circ \cdots \circ K^{\left(1\right)}\circ P\right)\left(a\right)$$

where $K^{\left(l\right)}$ is the *l*-th operator layer, and *L* represents the number of layers. First, we lift the input data a(x) to a higher dimensional space v(x) through the lifting operator $Q:R^{d_{\text{in}}}\to R^{d_{\text{hidden}}}$ as follows:

$$v=Q\left(a\right)\in R^{d_{\text{hidden}}}$$

Then, the lifted data are processed through the iterative process with Fourier layers $K^{\left(l\right)}$ as follows:

$$v_{l+1}=\sigma \left(W^{\left(l\right)}v^{\left(l\right)}+F^{-1}\left(R^{\left(l\right)}F\left(v^{\left(l\right)}\right)\right)\right)$$

where $R\in C^{M\times C_{\text{hidden}}\times C_{\text{hidden}}}$ is a Fourier domain weight matrix, $W\in R^{C_{\text{hidden}}\times C_{\text{hidden}}}$ is a weight matrix for transformation in the original spaces, and *M* is the number of low frequency modes. F, $F^{-1}$, and σ denote the FT, IFT, and an activation function, respectively. After the iterative processes, $v_{L}$ is projected to the output dimension function via the projection operator P:

$$u=P\left(v_{L}\right)\in R^{d_{\text{out}}}$$

The lifting operator and projection operator are performed as linear transformations on the channels.

### Performance metric calculation without numerical simulations

Our previous work demonstrates that the FoM can be calculated without requiring additional numerical simulations, leveraging advanced data augmentation techniques.^44^

$$\begin{array}{l} F_{1}=F_{0}+\frac{dF}{d\varepsilon_{x_{1}}}\Delta \varepsilon_{x_{1}}\\ F_{2}=F_{0}+\frac{dF}{d\varepsilon_{x_{1}}}\Delta \varepsilon_{x_{1}}+\frac{dF}{d\varepsilon_{x_{2}}}\Delta \varepsilon_{x_{2}}\\ \vdots \\ F_{k}=F_{0}+\sum_{i=1}^{k-1}\frac{dF}{d\varepsilon_{x_{i}}}\Delta \varepsilon_{x_{i}} \end{array}$$

If the initial performance metric $F_{0}$ is known, the updated performance metric $F_{1}$ can be calculated using our model by combining the inferred adjoint gradient $\frac{dF}{d\varepsilon }$ and the corresponding permittivity change at the same location. The permittivity change, Δϵ, refers to the variation in permittivity, such as that caused by adjusting the voltage in a tunable metasurface with liquid crystals.^45^^,^^46^

### Data acquisition

In this study, we simulate a tunable metasurface using the open-source finite-difference time-domain (FDTD) simulation tool Meep.^47^ The simulation is conducted in 2D to enhance computational efficiency while maintaining the metasurface’s core functionalities. The design region is structured as a barcode-like 1D array composed of LC. The LC’s permittivity is tunable within the range of 2.5–3.5 at 1,550 nm wavelength. In 2D simulations, we assume the permittivity change is uniform across the plane, which simplifies the model while effectively capturing the core optical behavior of the metasurface. However, in 3D implementations, this assumption may not hold as well since the complex alignment of the LC molecules in three dimensions can lead to non-uniform permittivity profiles. The data’s initial configuration is random, with 100 tunable pixels arranged within the design region. The design region has a width of 7.75 μm and a height of 775 nm. The source wavelength is set to the near-infrared (NIR) range, at 1,550 nm, with the surrounding environment assumed to be air. This simulation’s primary FoM is wave focusing, functioning as a lens. The tunable metasurface can focus light at various angles, so we set the focusing angle to 0°, ±5.73°, and ±11.31°. Additionally, we use 10,000 data points for each angle and split the data into training and test sets with an 8:2 ratio. For stabilized training, we scale the ground truth adjoint gradient values to the [0,1] range using empirically determined minimum and maximum values from the dataset, −0.239 and 0.388, respectively. The simulation domain size includes perfectly matched layers (PMLs) of 1 λ on all sides, with the simulation area extending 7.75 μm horizontally and 13.175 μm vertically. The source is placed 1 λ from the bottom of the simulation, and the design region is positioned 1 λ above the source. The focal point is set at a distance of 6.5 λ from the design region.

### Conclusion

We confirm that our surrogate solver meets all essential criteria for speed, accuracy, and optimization, effectively enhancing the scalability of the adjoint method. Furthermore, we empirically demonstrate that the surrogate solver provides highly accurate adjoint gradients for angles corresponding to untrained data, outperforming other conventional AI models. As a result, our surrogate solver emerges as a viable alternative to the adjoint method for beam focusing across multiple angles in tunable metasurfaces. The accuracy of the surrogate solver in predicting adjoint gradients and optimization outcomes closely matches that of the adjoint method, while offering a computational speedup of 887.5 times in inference. By combining exceptional computational speed with the ability to generalize to untrained targets, our surrogate solver opens new possibilities for addressing photonic design challenges. As a next step, we plan to experimentally validate the performance of our surrogate solver to ensure its robustness in real-world applications. Additionally, we aim to extend this work by applying the method to more complex photonic structures, such as CMOS image sensor (CIS) designs and large-scale photonic structures that require substantial computational resources. By combining exceptional computational speed, our surrogate solver has the potential to advance photonic design and other computationally demanding fields significantly.

### Limitations of the study

Surrogate solvers trained on a single wavelength often require datasets across multiple wavelengths for broadband simulations, making the process time-intensive. However, as shown in Figure 5, our model outperforms other AI methods in predicting untrained data.

To further improve accuracy, the use of the wave interpolation neural operator (WINO)^48^ could be beneficial. WINO takes discrete E-field data at specific wavelengths and interpolates the E-field for continuous wavelengths. However, since WINO is designed to predict the E-field rather than directly estimate the adjoint gradient, as we do in this study, additional study would be required to adapt it for this purpose.

Another limitation arises from the training data. For each angle, we utilized 10,000 data points, resulting in a total of 50,000 FDTD simulations. The number of FDTD simulations required for training may exceed the number needed for traditional adjoint optimizations. This issue could potentially be addressed by employing photonic data augmentation methods,^44^ which can significantly expand the training dataset by several hundredfold using only two simulations.

## Resource availability

### Lead contact

Requests for further information and resources should be directed to and will be fulfilled by the lead contact, Haejun Chung (haejun@hanyang.ac.kr).

### Materials availability

This study did not generate new materials.

### Data and code availability

• The entire dataset and codes used in this paper are deposited in the GitHub and available as of the date of publication.
• Any additional information required to reanalyze the data reported in this paper is available from the lead contact upon request.

## Acknowledgments

This work was supported by the 10.13039/501100003725National Research Foundation of Korea (NRF) grant (RS-2024-00338048 and RS-2024-00414119) funded by the Republic of Korea’s MSIT (Ministry of Science and ICT), under the Global Research Support Program in the Digital Field program (RS-2024-00412644) supervised by the 10.13039/501100010418IITP (Institute of Information and Communications Technology Planning & Evaluation); Culture, Sports and Tourism R&D Program through the 10.13039/501100006465Korea Creative Content Agency grant funded by the Ministry of Culture, Sports and Tourism in 2024 (RS-2024-00332210); Artificial Intelligence Graduate School Program (RS-2020-II201373, Hanyang University) supervised by the 10.13039/501100010418IITP; the artificial intelligence semiconductor support program to nurture the best talents (IITP(2024)-RS-2023-00253914) grant funded by the Korea government; and by the 10.13039/501100014188MSIT (RS-2023-00261368) grant funded by the Korea government.

## Author contributions

Conceptualization, C.K. and J.S.; methodology, C.K., J.S., and H.C.; investigation, C.K. and J.S.; writing – original draft, C.K., J.S., and H.C.; writing – review & editing, C.K., J.S., I.J., and H.C; funding acquisition, H.C.; supervision, H.C.

## Declaration of interests

The authors declare no competing interests.

## STAR★Methods

### Key resources table

**Table:** 

REAGENT or RESOURCE	SOURCE	IDENTIFIER
**Deposited data**		

Raw and analyzed data	This paper	https://github.com/nanophotonics-lab/Adjoint-FNO

**Software and algorithms**		

Python	Version 3.9.0	https://www.python.org/
Numpy	Version 1.26.4	https://numpy.org
Pytorch	Version 2.1.1	https://pytorch.org/
Meep	Version 1.28.0	https://github.com/NanoComp/meep
Autograd	Version 1.6.2	https://github.com/HIPS/autograd
Matplotlib	Version 3.8.4	https://matplotlib.org
Custom computer code	This paper	https://github.com/nanophotonics-lab/Adjoint-FNO

### Method details

#### Data collection

We conducted FDTD simulations using the open-source tool Meep to generate data for this study. The design region included 100 tunable LC pixels, with permittivity values ranging from 2.5 to 3.5 at a wavelength of 1550 nm. We focused on wave focusing as the primary FoM and simulated the metasurface to focus light at angles of 0°, ±5.73°, and ±11.31°. We generated 10,000 data points for each angle and divided the dataset into training and test sets with an 8:2 ratio.

#### Model construction

We employed an FNO to learn and predict adjoint gradients efficiently. We set a simple MLP as a baseline for performance comparison. Additionally, given that simulations governed by Maxwell’s equations are more influenced by nearby pixels, we included a 1D convolutional UNet as another baseline, which can incorporate a locality inductive bias.

The FNO comprises four layers, with 30 frequency modes in the Fourier layer and a channel dimensions 24. The scalar condition information, angles, was vectorized using sinusoidal time embedding^49^ and added to the input to incorporate conditional information. The MLP used for evaluation consists of four layers, with a channel dimension of 256, and the angle condition was concatenated to the input data. Similarly, the 1D convolutional UNet comprises four layers with a channel dimension of 32, and the angle condition was concatenated along the channel axis of the input data.

### Quantification and statistical analysis

For quantification and statistical analysis, we utilized Ubuntu 20.04 software and Python version 3.9.0, which run on a PC equipped with an AMD EPYC 7763 64-Core Processor and NVIDIA RTX 4090 GPU.

#### Figure of merit comparison between the adjoint method and our method

To evaluate the proposed method’s wavefront shaping performance, we visualized the residuals of the FoM values between structures optimized using adjoint gradients from the adjoint method and those optimized using adjoint gradients derived from the models (Figures 3B and 4).

The final residual results were calculated for the evaluation by averaging the FoM values across all angles used during training. The FoM values for each angle were normalized using the last iteration FoM value of the structure optimized by the adjoint gradient derived from the adjoint method. This normalization step is necessary because the range of FoM values can vary depending on the FoM criteria. Normalizing the values ensures that the averaged FoM values appropriately reflect the performance across all angles, providing a more balanced evaluation.

#### Accuracy of predicted adjoint gradients for untrained conditions

As discussed in this paper, when training on discrete conditions (angles), the inability to make accurate predictions for unseen conditions during training would necessitate retraining for those specific conditions. This requires substantial time and computational resources, posing a barrier to practical application. To evaluate the generalization performance, we assessed the normalized MAE of the predicted adjoint gradients not only for the angles used during training but also for those that were not included in the training process (Figure 5).
